# Hospital-Based Surveillance for Viral Hemorrhagic Fevers and Hepatitides in Ghana

**DOI:** 10.1371/journal.pntd.0002435

**Published:** 2013-09-19

**Authors:** Joseph Humphrey Kofi Bonney, Mubarak Osei-Kwasi, Theophilus Korku Adiku, Jacob Samson Barnor, Robert Amesiya, Chrysantus Kubio, Lawson Ahadzie, Stephan Ölschläger, Michaela Lelke, Beate Becker-Ziaja, Meike Pahlmann, Stephan Günther

**Affiliations:** 1 Virology Department, Noguchi Memorial Institute of Medical Research, University of Ghana, Legon, Ghana; 2 Department of Virology, Bernhard-Nocht-Institute for Tropical Medicine, Hamburg, Germany; 3 University of Ghana Medical School, Korle Bu, Ghana; 4 St. Theresa's Hospital, Nandom, Ghana; 5 West Gonja Hospital, Damongo, Ghana; 6 Disease Surveillance Department, Ghana Health Service, Accra, Ghana; U.S. Naval Medical Research Unit Six, United States of America

## Abstract

**Background:**

Viral hemorrhagic fevers (VHF) are acute diseases associated with bleeding, organ failure, and shock. VHF may hardly be distinguished clinically from other diseases in the African hospital, including viral hepatitis. This study was conducted to determine if VHF and viral hepatitis contribute to hospital morbidity in the Central and Northern parts of Ghana.

**Methodology/Principal Findings:**

From 2009 to 2011, blood samples of 258 patients with VHF symptoms were collected at 18 hospitals in Ashanti, Brong-Ahafo, Northern, Upper West, and Upper East regions. Patients were tested by PCR for Lassa, Rift Valley, Crimean-Congo, Ebola/Marburg, and yellow fever viruses; hepatitis A (HAV), B (HBV), C (HCV), and E (HEV) viruses; and by ELISA for serological hepatitis markers. None of the patients tested positive for VHF. However, 21 (8.1%) showed anti-HBc IgM plus HBV DNA and/or HBsAg; 37 (14%) showed HBsAg and HBV DNA without anti-HBc IgM; 26 (10%) showed anti-HAV IgM and/or HAV RNA; and 20 (7.8%) were HCV RNA-positive. None was positive for HEV RNA or anti-HEV IgM plus IgG. Viral genotypes were determined as HAV-IB, HBV-A and E, and HCV-1, 2, and 4.

**Conclusions/Significance:**

VHFs do not cause significant hospital morbidity in the study area. However, the incidence of acute hepatitis A and B, and hepatitis B and C with active virus replication is high. These infections may mimic VHF and need to be considered if VHF is suspected. The data may help decision makers to allocate resources and focus surveillance systems on the diseases of relevance in Ghana.

## Author Summary

Ghana is endemic for yellow fever and lies between two Lassa fever endemic areas — Guinea, Liberia, Sierra Leone, and Mali in the West, and Nigeria in the East. Ebola hemorrhagic fever has been documented in the neighboring Cote d'Ivoire. Thus, it is plausible that the latter VHFs also occur in Ghana, and there have been rumors of cases, which present like VHF in the north of the country. Our study aimed at verifying or disproving this suspicion. At 18 hospital-based study sites in the Central and Northern part of the country, samples from 258 patients with VHF symptoms were collected and tested for various VHF by PCR. As viral hepatitis is an important differential diagnosis of yellow fever, we also tested for several serological and molecular hepatitis markers. Rather surprisingly, VHFs were not detected, indicating that, even if they are endemic in the North of Ghana, they do not significantly contribute to hospital morbidity. However, a large fraction of patients showed markers of acute hepatitis A, and active hepatitis B and C. Children were mainly affected by hepatitis A, while adults were affected by hepatitis B and C. Hepatitis A and B are vaccine-preventable, and chronic hepatitis B and C are treatable diseases. Further efforts are needed to reduce the burden of these diseases in Ghana.

## Introduction

Viral hemorrhagic fevers (VHF) are acute viral diseases associated with bleeding, organ failure, and shock. The syndrome is caused by RNA viruses belonging to the families *Filoviridae* (Ebola and Marburg virus), *Arenaviridae* (Lassa virus), *Bunyaviridae* (Crimean-Congo hemorrhagic fever [CCHF] and Rift Valley fever [RVF] virus), and *Flaviviridae* (yellow fever [YF] virus). The case fatality rate depends on the causative virus and may be as high as 90% [1].

Several VHFs are endemic in West Africa, such as Lassa fever [2], Ebola hemorrhagic fever [3], CCHF [4], [5], RVF [6], and YF [7], [8]. Within the region, Ebola virus infection was documented so far only once in Cote d'Ivoire [3]. Lassa fever is endemic in the countries of Guinea, Sierra Leone, Liberia, Mali, and Nigeria [2], but has not been documented in Ghana. YF is endemic in Ghana [7], [8]. Since 1950, three major outbreaks — in 1969–70, 1977–80, and 1982–83 — affected the country and caused more than 400 deaths [9]. Whether other VHFs are endemic in Ghana, is not known. The list of differential diagnoses is long, because clinically, VHF is not easily distinguished from other febrile diseases in Africa. In particular, liver damage due to viral hepatitis may hardly be distinguished from YF. The presence of hepatitis B, C, and E virus in Ghana is documented by seroprevalence studies [10]–[19], while there is no published data on hepatitis A.

For several years, there have been anecdotic reports of cases presenting with VHF symptoms in the north of the country. The scarcity of reliable data on suspect cases is in part due to the lack of diagnostic tools and active surveillance systems. Therefore, we established PCR diagnostics for VHF at Noguchi Institute and conducted a hospital-based surveillance study to determine the etiology of illnesses presenting with VHF symptoms in the north of Ghana. In addition, viral hepatitis being an important differential diagnosis of hemorrhagic fevers was included in the study.

## Materials and Methods

### Ethics statement

The study was approved by the Institutional Review Board of the Noguchi Memorial Institute of Medical Research (NMIMR-IRB 003/07-08). All subjects provided written informed consent.

### Study area and subjects

**Figure 1:**
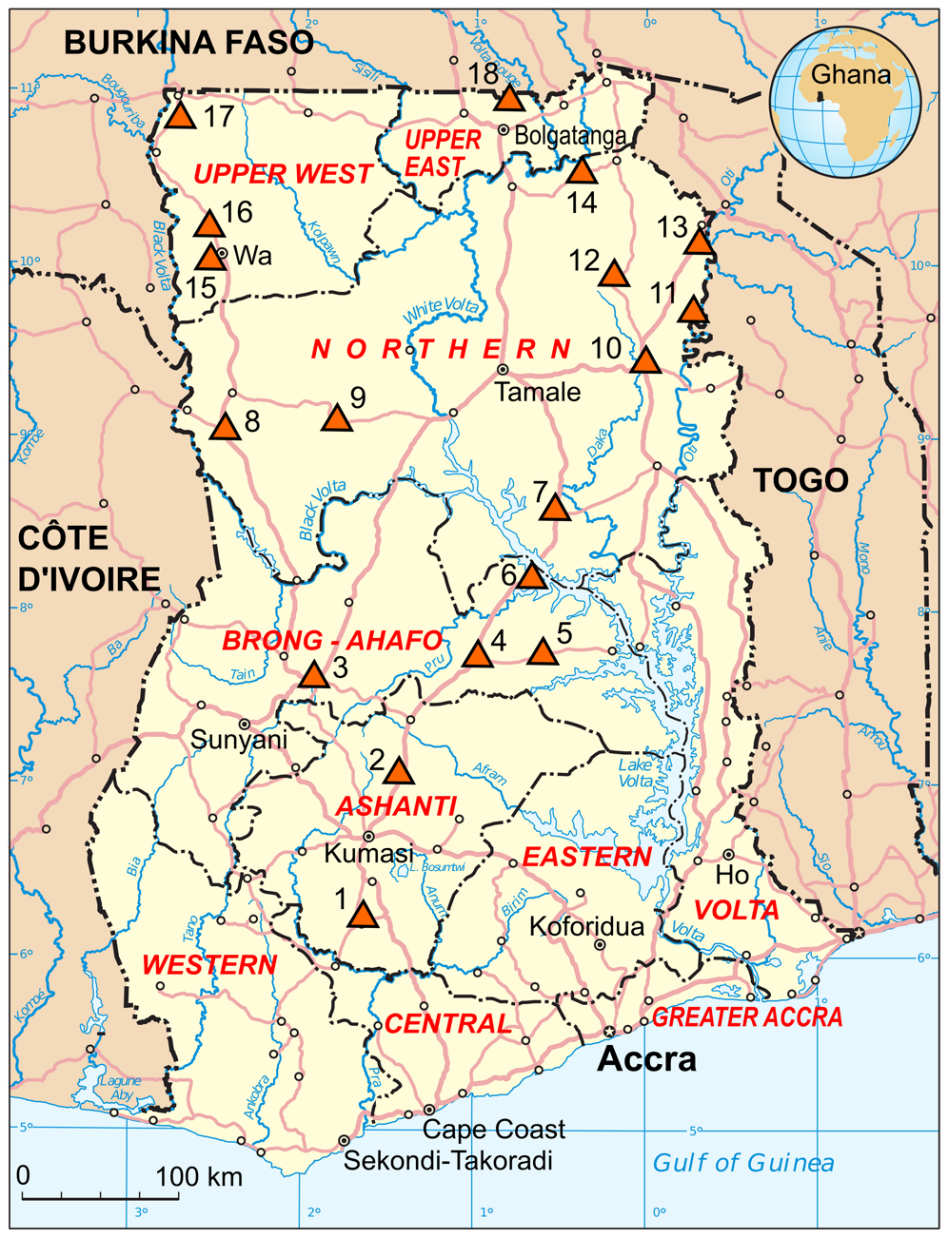
Map of Ghana showing the study sites (triangles). The names of the hospitals and regions (numbers of study participants in parentheses) are: 1. Obuasi District Hospital, Obuasi, Ashanti (n = 7). 2. Sekyere South Hospital, Sekyere South, Ashanti (n = 1). 3. Holy Family Hospital, Techiman, Brong-Ahafo (n = 5). 4. Atebubu District Hospital, Atebubu, Brong-Ahafo (n = 14). 5. Kwame Danso Health Center, Kwame Danso, Brong-Ahafo (n = 11). 6. Yeji Mathias Hospital, Yeji, Brong-Ahafo Region (n = 37). 7. Salaga Government Hospital, Salaga, Northern (n = 5). 8. Bole Health Center, Bole, Northern (n = 6). 9. West Gonja Hospital, Damongo, Northern (n = 35). 10. Yendi Municipal Hospital, Yendi, Northern (n = 6). 11. Saboba Medical Center, Saboba, Northern (n = 9). 12. Gushiegu District Hospital, Gushiegu, Northern (n = 31). 13. Chereponi Health Center, Chereponi, Northern (n = 1). 14. Nalerigu Baptist Hospital, Nalerigu, Northern (n = 12). 15. Wa Regional Hospital, Wa, Upper West (n = 2). 16. Kaleo Health Center, Kaleo, Upper West (n = 3). 17. Nandom St. Theresa's Hospital, Nandom, Upper West (n = 72). 18. Bongo Hospital, Bongo, Upper East (n = 1). Damango in the Northern region was included due to its proximity to Mole, the largest National Park in the country harboring a wide range of wildlife. Catholic mission hospitals serve Damango, Techiman, and Nandom, while Nalerigu and Kaleo have Baptist and Ahmadian mission hospitals, respectively. The other sites are government hospitals or health posts. Most of the areas are characterized by prolonged dry season and a short rainy season. The vegetation in the north is typical Sahel savannah with short trees including baobab, Shea butter trees, and long grass. Majority of the inhabitants in the Northern and the Upper West and East regions are subsistence farmers who grow maize cashew, millet, and groundnut and keep livestock. The three regions in the north are the poorest of the country. The Brong-Ahafo and Ashanti regions, however, are in the forest zone and major cocoa and timber producing areas. Most of the people live in multi-family compounds of dispersed settlements. The houses built largely of mud with either thatched, mud or iron roofing are common in most villages and towns in Northern Ghana. The map is based on a UN map. Source: UN Cartographic Section (http://www.un.org/Depts/Cartographic/english/about.htm).

The study was carried out from 2008 through 2011 at 18 hospitals in the Ashanti, Brong-Ahafo, Northern, Upper West, and Upper East regions in the Central and Northern sectors of Ghana (Fig. 1). In total, 285 patients were enrolled during the study period, of whom the first 27 patients were tested in a pilot activity on a reduced set of parameters. There was no pre-defined study group size. VHF patients often do not bleed, may present with a multitude of symptoms including jaundice, encephalopathy, and renal failure, and may be normo- or hypothermic in the terminal stage [1], [8], [20]–[23]. Therefore, a broad case definition was designed as a guideline for the local clinicians and health staff to screen and enroll the patients in the study sites. Criteria for including patients were severe illness with fever or history of fever and at least one of the following conditions: hemorrhage, jaundice, encephalopathy, renal involvement, absence of malaria, and lack of response to antibiotics and antimalarials. These criteria also include symptoms of severe liver disease due to viral hepatitis A, B, C, and E (jaundice, hepatic encephalopathy, bleeding due to impaired synthesis of coagulation factors, and renal failure due to hepatorenal syndrome). The principal investigators from Noguchi Institute regularly visited the study sites and trained local hospital staff on the case definition to minimize a selection bias due to different interpretations of the criteria.

### Sample collection

A blood specimen was taken and basic clinical and demographic data were recorded on the case investigation form. Serum was separated by centrifugation. If a centrifuge was not available, blood was kept in the refrigerator until there was complete retraction of the clot and serum could be removed. Serum was stored in the refrigerator for a maximum of one week at the study site and transported in a cool box with ice packs to the laboratory at Noguchi Memorial Institute. In the laboratory, sera were stored at −20°C.

### PCR assays

Viral nucleic acid was extracted from 140 µl serum using the QIAamp viral RNA kit (Qiagen). All PCR assays were performed in a volume of 25 µl with 2.5 µl or 5 µl nucleic acid extract as a template. Nested PCRs contained 1 µl PCR product of the first round RT-PCR as a template. Reagents, cycle numbers, primer sequences, target region, and amplicon length are shown in Table 1. Conventional RT-PCR assays for Lassa virus [24], RVF virus [25], flaviviruses including YF and dengue virus [26], CCHF virus [27], [28], and filoviruses including Ebola and Marburg virus [29] were performed at Noguchi Memorial Institute using a GeneAmp PCR System 2700 (Applied Biosystems). Testing for hepatitis A virus (HAV), hepatitis B virus (HBV), hepatitis C virus (HCV), and hepatitis E virus (HEV) was performed at Bernhard-Nocht-Institute. First, samples were tested using RealStar real-time PCR kits for HAV, HBV, HCV, and HEV (kindly provided by Thomas Laue, altona Diagnostics, Germany) on a LightCycler 480 (Roche) according to the manufacturer's instructions. In addition, conventional PCR assays were performed using a Primus25advanced thermocycler (PeqLab, Germany): HBV PCR [30] and two nested HEV RT-PCR assays, ORF2-457 PCR [31] and ORF2/3-137 PCR [32], were done on all samples, while HAV [33], [34] and HCV [35] RT-PCR assays were done only on samples positive for these viruses in the real-time RT-PCR.

**Table 1:** PCR assays used in the study.

Virus	Reagents; cycles; primers (sequence 5′-3′)	Target region	Amplicon length [base pairs]	Reference
Lassa	OneStep RT-PCR kit (Qiagen); 45 cycles; 36E2 (ACCGGGGATCCTAGGCATTT), LVS-339-rev (GTTCTTTGTGCAGGAMAGGGGCATKGTCAT)	5′UTR/GPC gene	320	[24]
RVF	OneStep RT-PCR kit (Qiagen); 45 cycles; RVS (AAAGGAACAATGGACTCTGGTCA), RVAs (CACTTCTTACTACCATGTCCTCCAAT)	G2 gene	95	[25]
Flavi^a^	OneStep RT-PCR (Qiagen); 45 cycles; VD8 (GGGTCTCCTCTAACCTCTAG), EMF1 (TGGATGACSACKGARGAYATG)	NS5/3′UTR	500–700	[26]
CCHF	OneStep RT-PCR kit (Qiagen); 45 cycles; CC1a_for (GTGCCACTGATGATGCACAAAAGGATTCCATCT), CC1b_for (GTGCCACTGATGATGCACAAAAGGATTCTATCT), CC1c_for (GTGCCACTGATGATGCACAAAAGGACTCCATCT), CC1a_rev (GTGTTTGCATTGACACGGAAACCTATGTC), CC1b_rev (GTGTTTGCATTGACACGGAAGCCTATGTC), CC1c_rev (GTGTTTGCATTGACACGGAAACCTATATC)	N gene	280	[28]
CCHF	OneStep RT-PCR kit (Qiagen); 45 cycles; RWCF (CAAGGGGTACCAAGAAAATGAAGAAGGC), RWCR (GCCACAGGGATTGTTCCAAAGCAGAC)	N gene	180	[27]
Filo^b^	OneStep RT-PCR kit (Qiagen); 45 cycles; FiloA2.2 (AAGCCTTTCCTAGCAACATGATGGT), FiloA2.3 (AAGCATTCCCTAGCAACATGATGGT), FiloA2.4 (AAGCATTTCCTAGCAATATGATGGT), Filo B (ATGTGGTGGGTTATAATAATCACTGACATG), Filo B-Ra (GTGAGGAGGGCTATAAAAGTCACTGACATG)	L gene	290	[29]
HBV	Platinum Taq polymerase (Invitrogen); 40 cycles; HBV-2263(+) (TTCGGAGTGTGGATTCGCACTCCT), HBV-2958(−) (GTTGGGATTGAAGTCCCAATCTGGAT)	P gene	730	[30]
HEV	First-round: Superscript II One Step RT-PCR kit (Invitrogen); 40 cycles; HE040 (CCCTTRTCCTGCTGAGCRTTCTC), HE044 (CAAGGHTGGCGYTCKGTTGAGAC); Nested: Platinum Taq polymerase (Invitrogen); 25 cycles; HE110-2 (mixture of GYTCKGTTGAGACCTCYGGGGT, GYTCKGTTGAGACCACGGGYGT, GYTCKGTTGAGACCTCTGGTGT), HE041 (TTMACWGTCRGCTCGCCATTGGC)	ORF2	460	[31]
HEV	First-round: Superscript II One Step RT-PCR kit (Invitrogen); 35 cycles; HE361 (GCRGTGGTTTCTGGGGTGAC), HE364 (CTGGGMYTGGTCDCGCCAAG); Nested: Platinum Taq polymerase (Invitrogen); 25 cycles; HE363 (GMYTGGTCDCGCCAAGHGGA), HE366 (GYTGATTCTCAGCCCTTCGC)	ORF2/ORF3	140	[32]
HAV	OneStep RT-PCR kit (Qiagen); 40 cycles; 2870 (GACAGATTCTACATTTGGATTGG), 3381 (CCATTTCAAGAGTCCACACACT)	VP1/2A junction	510	[33], [34]
HCV	OneStep RT-PCR kit (Qiagen); 45 cycles; Pr3 (TATGAYACCCGCTGYTTTGACTC), Pr4 (GCNGARTAYCTVGTCATAGCCTC)	NS5B	380	[35]

a Flaviviruses including YF and dengue virus.

b Filoviruses including Ebola and Marburg virus.

### Serological assays

Serological tests for HAV, HBV, and HEV were performed in 96-well ELISA format using commercially available kits according to the manufacturer's instructions: HAV IgM ELISA (DRG, Germany), HBsAg one Version Ultra (Dia.Pro, Italy), HBc IgM (Dia.Pro, Italy), and RecomWell HEV IgG and IgM (Mikrogen, Germany).

### Clinical chemistry

Serum levels of total protein, albumin, total bilirubin, aspartate aminotransferase (AST), alanine aminotransferase (ALT), lactate dehydrogenase (LDH), amylase, creatinine, and urea nitrogen were determined with a Spotchem EZ SP-4430 analyzer (Arkray, Japan) at room temperature. Samples had been stored for ≥3 years at −20°C before analysis. Reference values were defined according to Kratz et al. [36].

### Sequencing and phylogenetic analysis

PCR products generated in the conventional PCRs were sequenced on both stands using the PCR primers. The sequences were assembled using Lasergene software (DNASTAR) and automated base calling was proofread by visual inspection of the electropherograms. Phylogenetic analysis included the novel sequences of HAV (VP1/2A junction; n = 8), HBV (N terminal P protein region; n = 43), and HCV (NS5B region; n = 10) as well as sequences available from GenBank by December 2012. Three categories of GenBank entries were retrieved: (i) sequences with close relationship to the novel sequences as determined by BLAST search, (ii) sequences from Ghana, and (iii) representative sequences of genotypes and sub-genotypes [33], [37], [38]. The program jModelTest 0.1.1 [39] identified the general time-reversible model of sequence evolution with a gamma distribution of among-site nucleotide substitution rate variation (GTR+gamma) as the substitution model that best describes the data in the nucleotide sequence alignment for HAV (37 taxa, 467 sites), HBV (117 taxa, 684 sites), and HCV (85 taxa, 337 sites). Phylogenies were inferred by the Bayesian Markov Chain Monte Carlo method implemented in BEAST software [40]. Initially, BEAST was run with the uncorrelated relaxed lognormal clock to measure the ucld.stdev parameter which gives an indication of how clock-like the data is (HAV, ucld.stdev 0.16; HBV, ucld.stdev 0.63; HCV, ucld.stdev 0.31) [41]. The final analysis was performed using the parameters: GTR+gamma; constant population size; strict clock for HAV, relaxed exponential clock for HBV, and relaxed lognormal clock for HCV; 10^7^ steps with sampling every 10^4^th step; and 3 independent runs combined (effective sampling size >200 for all parameters).

### Statistical analysis

Quantitative variables were analyzed using non-parametric descriptive statistics and statistical tests. Statistical comparison of unpaired groups was performed for continuous parameters with the Mann–Whitney U test and for frequencies with two-tailed Fisher's Exact test. A critical p value of 0.01 was considered appropriate, given that several tests were performed on the data set. If a study participant had missing data for a specific variable, he/she was excluded from the analysis of this variable, but not of the other variables. Relative frequencies (e.g. in %) for a variable were calculated with the denominator: group size minus number of participants with missing data for this variable.

### Accession numbers

A representative set of 6 HAV, 28 HBV, and 10 HCV sequences has been sent to GenBank and assigned the accession nos. KC632110–KC632153.

## Results

### Virological findings

The project started in 2008 with a pilot study involving 27 patients. VHF was not detected, but 7 (26%) patients had acute hepatitis B or an exacerbation of chronic hepatitis B as evidenced by presence of HBV DNA, HBsAg, and anti-HBc IgM or seroconversion to HBsAg/HBV DNA-positive. Therefore, the design of the main study was adjusted to incorporate a wider range of tests for viral hepatitis.

**Figure 2:**
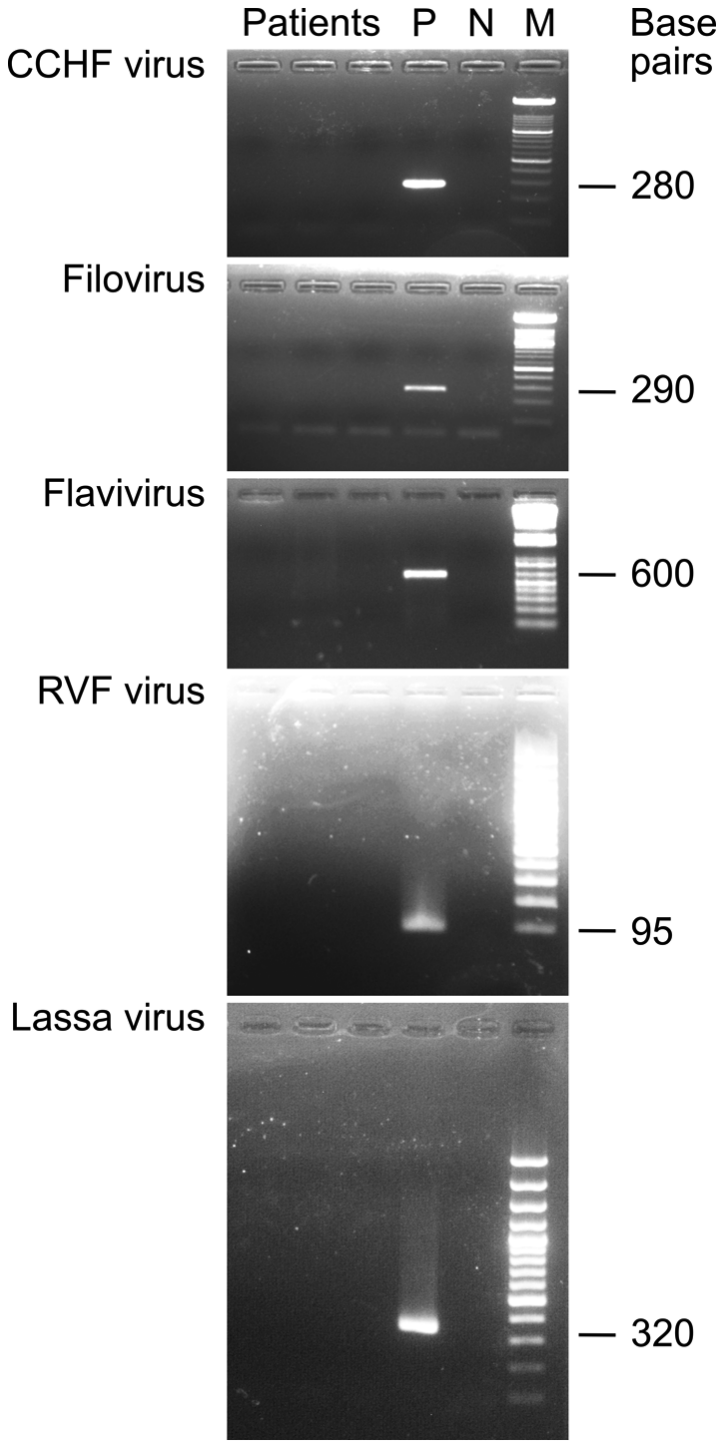
Representative results of VHF RT-PCR screening. Positive (P) and negative controls (N) were included in each PCR assay. The expected length of the PCR products is indicated right to the 100-base pair marker (M).

The main study was conducted from 2009 to 2011 and included 258 patients from 18 study sites. The number of patients per site ranged from 1 to 72 (legend to Fig. 1). The male-to-female ratio was 68∶32 and median age was 23.5 years. Fever was reported in 74%, jaundice in 48%, and hemorrhage in 13% of the cases. None of the patients tested positive by PCR for Lassa virus, filoviruses including Ebola and Marburg virus, flaviviruses including dengue and YF virus, RVF virus, and CCHF virus (Fig. 2). However, 105 (41%) of the patients had evidence of HBV infection: 100 (39%) were HBsAg-positive and 59 (23%) were HBV DNA-positive, of whom 56 (22%) were positive for both markers (Table 2). Anti-HBc IgM was detected in 21 (8.1%) of HBV DNA and/or HBsAg-positive patients indicating acute or fulminant hepatitis B or severe exacerbation of chronic hepatitis B. Presumed chronic hepatitis B with active virus replication was diagnosed in another 37 (14%) patients, who were positive for HBsAg and HBV DNA, but negative for anti-HBc IgM. In total, 26 (10%) patients had evidence of acute hepatitis A: 25 (9.7%) were anti-HAV IgM-positive and 19 (7.4%) were HAV RNA-positive, of whom 18 (7.0%) were positive for both markers. HCV RNA was detected in 20 (7.8%) patients, indicating hepatitis C with active virus replication. Serological HEV markers were found in 10 (3.9%) patients showing anti-HEV IgM and 15 (5.8%) patients showing anti-HEV IgG. However, none showed both markers. In addition, none of the study patients was positive for HEV RNA upon testing with 3 different PCR assays.

**Table 2:** Demographic data and symptoms of patients.

Variable	No diagnosis (n = 157)	Acute hepatitis A (n = 26)^a^	Acute hepatitis B (anti-HBc IgM +ve) (n = 21)^b^	Presumed chronic hepatitis B (anti-HBc IgM −ve) (n = 37)^c^	Presumed chronic hepatitis C (n = 20)^d^
Male/female, %	64/36	77/23	71/29	76/24	74/26
Missing data, n	6	–	4	–	1
Age in years, median (Q25–Q75)	18 (4–30)^e^	5 (3–7)^e^	30 (18–35)^e^	30 (26–35)^e^	32 (25–49)^e^
Missing data, n	5	1	5	–	1
Region, n (%)					
Brong-Ahafo & Ashanti	49 (31)	10 (38)	5 (24)	9 (24)	3 (15)
Northern	50 (32)^f^	9 (35)	11 (52)^f^	26 (70)^f^	11 (55)
Upper West & East	58 (37)^g^	7 (27)	5 (24)^g^	2 (5)^g^	6 (30)
Symptoms, n (%)					
Fever	115 (73)	21 (81)	17 (81)	30 (81)	10 (50)
Jaundice	61 (39)^h^	20 (77)^h^	17 (81)^h^	19 (51)	7 (35)
Hemorrhage	23 (15)	3 (12)	2 (10)	3 (8)	2 (10)
Missing data, n	1	–	–	–	–

Statistical analysis of the data: Each sub-group with diagnosis was tested vs. the sub-group without diagnosis for all quantitative variables and categories of a qualitative variable. Statistically significant differences are indicated by footnotes; all other differences were not significant.

Abbreviations: Q25, lower quartile, 25% of the data lie below this value; Q75, upper quartile, 75% of the data lie below this value.

a 18 patients were positive for anti-HAV IgM and HAV RNA; 7 patients were positive for anti-HAV IgM; 1 patient was positive for HAV RNA. Co-infections: 1 patient was positive for HBV DNA and HBsAg.

b 19 patients were positive for anti-HBc IgM, HBV DNA, and HBsAg; 2 patients were positive for anti-HBc IgM and positive for HBV DNA in two different PCR assays, but negative for HBsAg. Co-infections: 1 patient was HCV RNA positive.

c All patients were positive for HBV DNA and HBsAg, but negative for anti-HBc IgM. Co-infections: 1 patient was anti-HAV IgM positive; 1 patient was HCV RNA positive.

d All patients were HCV RNA positive. Co-infections: 1 patient was positive for anti-HBc IgM, HBV DNA, and HBsAg; 1 patient was positive for HBV DNA and HBsAg.

e p = 0.001, no diagnosis vs. acute hepatitis A; p = 0.0001, no diagnosis vs. chronic hepatitis B; p = 0.00004, no diagnosis vs. acute & chronic hepatitis B combined; p = 0.005, no diagnosis vs. hepatitis C.

f p<0.0001, no diagnosis vs. chronic hepatitis B; p<0.0001, no diagnosis vs. acute & chronic hepatitis B combined.

g p<0.0001, no diagnosis vs. chronic hepatitis B; p = 0.0004, no diagnosis vs. acute & chronic hepatitis B combined.

h p = 0.0005, no diagnosis vs. acute hepatitis A; p = 0.0003, no diagnosis vs. acute hepatitis B.

### Demographic and disease-related findings

Hepatitis A mainly affected children with a median age of 5, while hepatitis B and C mainly affected young adults with a median age around 30 years (Table 2). Compared to patients without diagnosis (median age 18 years), patients with hepatitis A were significantly younger (p = 0.001), and those with hepatitis B and C were significantly older (p = 0.00004 and p = 0.005, respectively). Patients with hepatitis B more frequently originated from the Northern region (p<0.0001) and less frequently from the Upper West & East regions (p = 0.0004) compared to patients without diagnosis (Table 2). The only symptom that showed a statistically significant association with a diagnostic category was jaundice; it was about twice as frequent among patients with acute hepatitis A or B than among patients without diagnosis (p = 0.0005 and p = 0.0003, respectively) (Table 2).

To provide additional evidence for an association of the virological diagnoses with the disease observed in the patients, clinical chemistry parameters were measured retrospectively for a subset of patients (n = 67) with a diagnosis of viral hepatitis (Table 3). Although the prolonged storage at −20°C and the freeze-thaw cycles predictably led to some loss of serum enzyme activities [42], [43], pathological changes were seen in most patients. AST and LDH were elevated in the majority of hepatitis cases. ALT, which is particularly sensitive to storage at −20°C [43], was elevated in a large fraction of patients with acute hepatitis A and B. Albumin was specifically decreased (relative to total protein) in most patients, while total bilirubin was elevated in particular in patients with acute hepatitis. The latter corresponds to the frequent finding of jaundice in this group. Between 60 and 100% of hepatitis patients showed 2 or more pathological values for parameters that indicate liver disease, namely albumin, total bilirubin, AST, ALT, and LDH (Table 3). In some patients, minor elevations of amylase, creatinine, and urea nitrogen were found. Overall, the clinical chemistry data show that the majority of patients with viral hepatitis has biochemical evidence of liver disease.

**Table 3:** Clinical chemistry data for a subset of patients with hepatitis A, B, and C.

		% Patients with pathological finding (mean of pathological values [mg/dl or U/l])^a^							
Parameter	Pathological range^b^	Acute hepatitis A (n = 11)		Acute hepatitis B (anti-HBc IgM +ve) (n = 13)		Presumed chronic hepatitis B (anti-HBc IgM −ve) (n = 30)		Presumed chronic hepatitis C (n = 13)	
Total protein	>8.0 g/dl	55		15		20		31	
Total protein	<5.5 g/dl	18		23		33		15	
Albumin	<3.5 g/dl	55		92		77		92	
Total bilirubin	>1.0 mg/dl	45	(14)	62	(7.5)	30	(4.2)	15	(8.5)
AST	>35 U/l	91	(369)	85	(630)	50	(258)	77	(278)
ALT	>35 U/l	36	(194)	46	(303)	7	(169)	8	(1838)
LDH	>190 U/l	55	(737)	62	(839)	57	(519)	85	(1477)
Amylase	>180 U/l	27	(279)	15	(348)	3	(256)	–	–
Creatinine	>1.5 mg/dl	27	(2.9)	8	(3.4)	7	(2.0)	8	(2.4)
Urea nitrogen	>20 mg/dl	–	–	31	(40)	20	(36)	38	(34)
≥2 Pathological findings^c^		73		100		60		85	

Inclusion in this analysis depended on the availability of sufficient amount of serum. Statistical analysis was not performed. There are no missing values.

Abbreviations: AST, aspartate aminotransferase; ALT, alanine aminotransferase; LDH, lactate dehydrogenase.

a Samples had been stored for ≥3 years at −20°C before analysis, which predictably leads to a certain loss of enzyme activity in serum, in particular of ALT [42], [43]. Therefore, the data shown in the table tend to underestimate the true values. For selected parameters, the mean of all values in the pathological range are shown in parentheses. Units and pathological range are defined in the second column.

b Reference values were taken from Kratz et al. [36].

c Percentage of patients showing 2 or more pathological values for parameters that may indicate liver disease: albumin, total bilirubin, AST, ALT, and LDH.

### Sequence analysis and phylogeny

**Figure 3:**
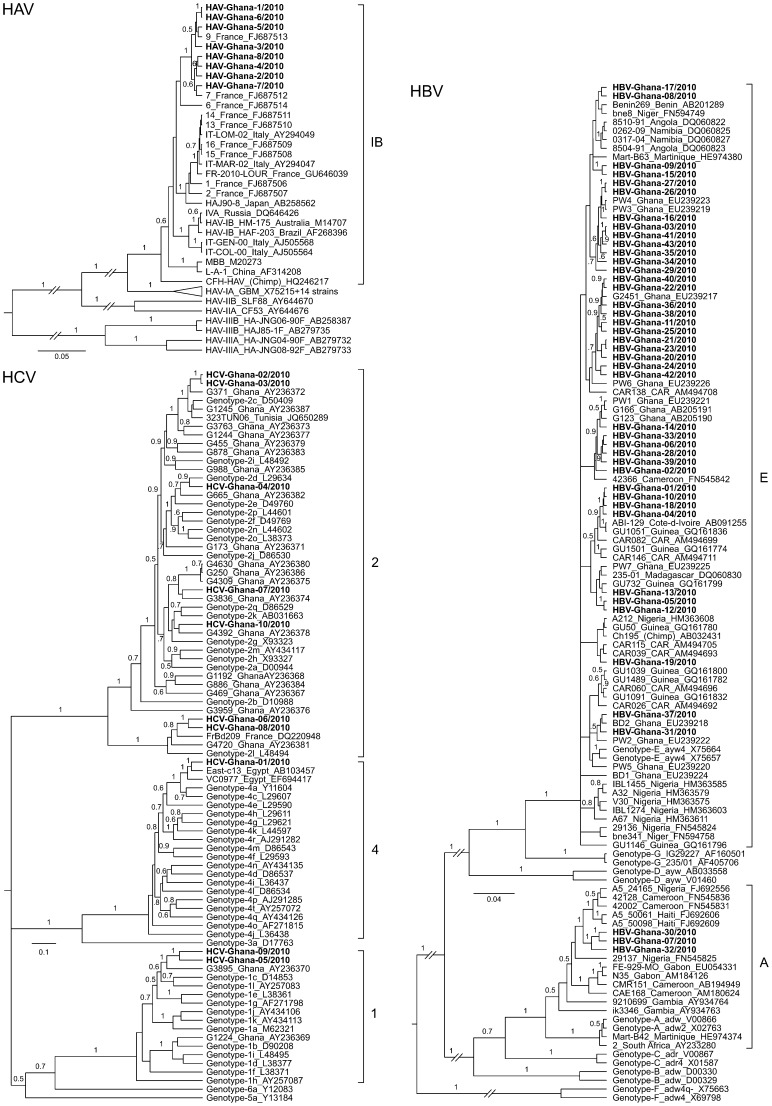
Phylogenetic analysis of HAV, HBV, and HCV sequences. The alignments to reconstruct the phylogenies included the novel sequences of HAV (467 nucleotides of VP1/2A junction), HBV (684 nucleotides of N terminal P protein region), and HCV (337 nucleotides of NS5B region) as well as sequences available from GenBank by December 2012. The latter are identified by GenBank accession numbers. Sequences of this study are highlighted in boldface (GenBank accession no. KC632110–KC632153). Primers used for amplification and sequencing are shown in Table 1. Virus genotypes are indicated with the strains or right to the tree. If available, the geographic origin of a strain is indicated as well. Posterior probability values are shown on the branches if ≥0.5. Some branches with low support values were collapsed for clarity of presentation.

To determine the genotype of HAV, HBV, and HCV circulating in the study area, the PCR products were sequenced and subjected to phylogenetic analysis (Fig. 3). The Ghanaian HAV strains are closely related to each other and form within genotype IB a separate clade (posterior probability value of 1), which also includes strains from France. The HBV strains from Ghana belong to genotype A and E. The genotype E strains are scattered over the phylogenetic tree and are related to other genotype E strains from West Africa. However, due to the close relationship among all genotype E viruses, the phylogenetic relationships within this clade could not be resolved. The genotype A strains from Ghana show a well-supported sister relationship (posterior probability values of 1) with strains from Nigeria, Cameroon, and Haiti, which have previously been classified as sub-genotype A5. The HCV strains belong to genotypes 1, 2, and 4, are highly divergent, and difficult to classify into sub-genotypes. The Ghanaian genotype 4 strain is related to sub-genotypes 4a and 4c and shows a close relationship to strains from Egypt. Within genotype 2, two strains are related to sub-genotype 2c, one is related to sub-genotype 2d, and two are related to sub-genotype 2l. The remaining two genotype 2 strains could not be sub-classified. Similarly, the sub-genotype of the two strains within genotype 1 could not be determined. Genotype 1 and 2 strains are somewhat related to previously described HCV strains from Ghana [17].

## Discussion

This study was conducted to determine if VHF or other infectious diseases presenting with similar symptoms are of medical importance in Central and Northern areas of Ghana. The main conclusion from our data is that VHF hardly contributes to hospital morbidity in the study area indicating a low incidence of severe VHF. This does not prove that VHF is absent and in fact, YF is endemic in the country and cases may be seen at any time. In addition, mild VHF cases may not attend the hospital. Instead of VHF, a high incidence of viral hepatitis was found.

The case definition to screen for potential VHF patients was intentionally broad. On the one hand, clinical complications of VHF can mimic other diseases [1]. For example, renal failure is a frequent complication in Lassa patients [20], central nervous system disturbance such as seizures or coma is frequent in Lassa fever and RVF [21], [22], and hepatic injury with jaundice is typical for RVF and YF [8], [23]. On the other hand, the pathognomonic bleeding is rare in some VHF [1] and even fever cannot be considered a *conditio sine qua non*, as patients presenting in the terminal stage of Lassa fever — and probably other VHFs as well — are often normo- or hypothermic [20]. In the end, the broad case definition facilitated the sampling of patients with other serious diseases, including acute and chronic viral hepatitis.

Whether the diagnosed HAV, HBV, or HCV infections actually caused the clinical symptoms is difficult to prove. As the study was focused on diagnostic aspects, only minimal clinical information was collected and additional invasive or non-invasive diagnostic procedures were not performed. However, the retrospective analysis of clinical chemistry parameters provides some clues. For patients positive for anti-HAV IgM and/or HAV RNA or anti-HBc IgM plus HBV DNA, there is little doubt that the clinical symptoms are due to HAV or HBV infection, respectively. Anti-HBc IgM is a classical marker of acute or fulminant hepatitis B, but may also indicate an acute exacerbation of chronic hepatitis [44], [45]. Anti-HAV IgM and presence of HAV RNA are diagnostic markers of acute hepatitis A. Liver damage in our hepatitis A patients and anti-HBc IgM-positive hepatitis B patients is evidenced by jaundice as well as elevated AST, ALT, LDH, and bilirubin levels in serum. The hemorrhage reported in a few patients might be interpreted as a sign of liver failure, although this speculation cannot be supported by additional data.

A large fraction of study patients showed HBsAg and HBV DNA without being anti-HBc IgM positive, suggesting chronic hepatitis B with active virus replication. The clinical chemistry data show that about 60% of these patients had liver damage (≥2 pathological values for albumin, total bilirubin, AST, ALT, and LDH). Similarly, about 85% of patients with active HCV infection, as evidenced by virus detection, had biochemical evidence of liver damage (≥2 pathological values). In particular, AST and LDH levels were elevated and albumin levels were decreased in both groups. Thus, it is plausible that a large fraction of anti-HBc IgM-negative hepatitis B patients and hepatitis C patients attended the hospital due to the related liver disease. However, superinfection by another pathogen may not be excluded as cause of the disease.

Acute hepatitis A and B may lead to fulminant liver failure in a small fraction of patients [46]–[49] and chronic hepatitis B and C are the worldwide leading causes of liver cirrhosis and hepatocellular carcinoma [50]. Reports from other West African countries confirm that hepatitis B and C are among the major causes of hepatocellular carcinoma, liver cirrhosis, and fulminant hepatic failure in the region [51]–[55], which is supported by our data. Hepatitis B is already part of the Expanded Program on Immunization in Ghana. Each child should receive three doses of DPT-HepB-Hib (pentavalent) vaccine formulation at 6, 10, and 14 weeks after birth (http://www.afro.who.int/en/ghana/country-programmes/3215-expanded-program-of-immunisation-epi.html). An immunization coverage survey conducted in 2008 showed a national coverage for the third dose (Penta 3) of 72% (range 40 to 89% at district level) [56]. Thus, the hepatitis B incidence is expected to decrease in the long term. In spite of this progress, the implementation in clinical practice of established treatment options for hepatitis B and C [57], [58] should be a strategic goal to reduce morbidity and mortality from both infections [50]–[55].

The vast majority of hepatitis A patients were young children. This is consistent with the known epidemiology of this infection. HAV is transmitted by the fecal-oral route, e.g. via contaminated food or water. In the developing world, infections are most frequently acquired during early childhood, resulting in a high proportion of adults that are immune to HAV [59].

The prevalence of HBsAg carriers among blood donors in the Northern and Ashanti regions is 11% [10], [11], [18], [19]. The HBsAg prevalence increases in Ghana after birth up to 20–35% in individuals aged 11–15 years and then decreases with age, while the percentage of anti-HBc, a marker of past or present HBV infection, steadily increases up to 70 and 90% in individuals aged 20 and 40 years, respectively [10], [11], [16], [19]. This pattern suggests an acquisition of HBV infection with advancing age predominantly through horizontal transmission in childhood [16]. The epidemiology of HCV is similar, though not identical with HBV. The HCV seroprevalence in the Ashanti region ranges from 3 to 10% [11], [14], [18]. It is already high in children and does not show a clear age-dependent increase [11], [14], implying that many HCV infections occur during childhood. In summary, a large percentage of HBV and HCV-infected individuals in Ghana carry the virus since childhood and presumably develop overt symptoms of liver disease in adulthood. This epidemiology is consistent with the higher age of our hepatitis B and C patients; half of them were older than 30 years. However, no correlation between HBV and HCV markers was previously found in Ghana [11], which is in agreement with the low frequency of HBV/HCV co-infections in our study.

The incidence of hepatitis B in the study hospitals of the Northern region and the Upper West & East regions was higher and lower, respectively, relative to the control group of patients without diagnosis. We have no good explanation for this finding. The three regions are the poorest of the country and comparable with respect to many indicators [60] (see also legend to Fig. 1). A close inspection of the HBV phylogenetic tree reveals one genotype E cluster that nearly exclusively comprises sequences from the Northern region (Fig. 3, HBV-Ghana sequences no. 40, 22, 36, 38, 11, 25, 21, 23, 20, and 24 from top to bottom), while all other clusters showed no geographic pattern. Whether this Northern region-specific lineage is associated with a higher incidence remains to be studied. A sampling bias due to differences in the patient selection procedure in the various study sites may also be taken into account.

A few patients showed serological markers of HEV infection. However, all PCR assays were negative, HEV immunoassays are prone to false reactivity [61], [62], and none of the patients showed both IgM and IgG. Usually, both serological markers and HEV RNA are present during acute infection ([62], [63] and validation data for the test used in this study [64]). Therefore, we conclude that acute HEV infection does not significantly contribute to hospital morbidity in the study area.

Though a minor aspect of our study, the phylogenetic analysis revealed some peculiarities of HAV, HBV, and HCV strains circulating in Ghana. All HAV strains are closely related to each other and belong to genotype IB, though the patients originate from Ashanti, Brong-Ahafo, Northern, or Upper West region. This suggests that a predominant HAV strain circulates in large parts of Ghana. In agreement with previous reports from Ghana and other West African countries [12], [13], the HBV strains of our study belong to genotype A and E. The genotype A strains also cluster with other strains from West Africa and may belong to sub-genotype A5. The HCV sequences reported here and previously from Ghana and other West African countries show that the strains in the region are extremely diverse, belong to various genotypes (1, 2, and 4) and sub-genotypes, and often cannot be classified into sub-genotypes, as they are too divergent from the reference strains [14], [17], [65], [66]. Further studies are warranted to clarify the interesting molecular epidemiology of HCV in Ghana.

## Supporting Information

Checklist S1 STROBE checklist.
(PDF)
