# Supplementary material for: Hospital-Based Surveillance for Viral Hemorrhagic Fevers and Hepatitides in Ghana
Source: PLoS Negl Trop Dis. 2013 Sep 19;7(9):e2435. doi: 10.1371/journal.pntd.0002435 (PMC3777898; doi:10.1371/journal.pntd.0002435)
Supplement: Checklist S1 — STROBE checklist. (PDF) [file pntd.0002435.s001.pdf]

Checklist S1: STROBE Statement—Checklist of items that should be included in reports of ***cross-sectional studies***

	Item No	Recommendation	Implementation in the study and comments
**Title and abstract**	1	(*a*) Indicate the study's design with a commonly used term in the title or the abstract	YES (hospital-based)
		(*b*) Provide in the abstract an informative and balanced summary of what was done and what was found	YES (region, study subjects, diagnostic tests, and relevant findings are provided)
**Introduction**			
Background/ rationale	2	Explain the scientific background and rationale for the investigation being reported	YES (situation in West Africa and Ghana is explained)
Objectives	3	State specific objectives, including any prespecified hypotheses	YES (to detect VHF or other severe infections in North Ghana)
**Methods**			
Study design	4	Present key elements of study design early in the paper	YES (hospital-based with laboratory investigation)
Setting	5	Describe the setting, locations, and relevant dates, including periods of recruitment, exposure, follow-up, and data collection	YES (study sites, period of study and means of data collection are provided)
Participants	6	(*a*) Give the eligibility criteria, and the sources and methods of selection of participants	YES (inclusion criteria and hospital staff selecting study subjects are described)
Variables	7	Clearly define all outcomes, exposures, predictors, potential confounders, and effect modifiers. Give diagnostic criteria, if applicable	YES (outcome is defined by laboratory testing; assays and diagnostic criteria are described in Methods and Results)
Data sources/ measurement	8*	For each variable of interest, give sources of data and details of methods of assessment (measurement). Describe comparability of assessment methods if there is more than one group	YES (methods of collection of data in hospital and laboratory are described; there is only one study group)
Bias	9	Describe any efforts to address potential sources of bias	YES (efforts to reduce selection bias are described in Methods)
Study size	10	Explain how the study size was arrived at	YES (no predefined group size)
Quantitative variables	11	Explain how quantitative variables were handled in the analyses. If applicable, describe which groupings were chosen and why	YES (non-parametric descriptive statistics and statistical tests)
Statistical methods	12	(*a*) Describe all statistical methods, including those used to control for confounding	YES (separate paragraph in Methods; Mann–Whitney U and Fisher's Exact test)
		(*b*) Describe any methods used to examine subgroups and interactions	YES (subgroups are defined according to diagnostic findings in the Results)
		(*c*) Explain how missing data were addressed	YES (patients with missing data were excluded from the analysis of this specific variable)
		(*d*) If applicable, describe analytical methods taking account of sampling strategy	Not applicable
		(*e*) Describe any sensitivity analyses	Not applicable
**Results**			
Participants	13*	(a) Report numbers of individuals at each stage of study—eg. numbers potentially eligible, examined for eligibility, confirmed eligible, included in the study, completing follow-up, and analysed	YES (numbers included and analyzed in the study are reported)

		(b) Give reasons for non-participation at each stage	Not applicable (only one stage, no follow up)
		(c) Consider use of a flow diagram	Not applicable
Descriptive data	14*	(a) Give characteristics of study participants (eg. demographic, clinical, social) and information on exposures and potential confounders	YES (absolute and relative frequencies, median, and quartiles in Results and Table 2)
		(b) Indicate number of participants with missing data for each variable of interest	YES (in Table 2)
Outcome data	15*	Report numbers of outcome events or summary measures	YES (outcome = diagnosis; numbers of subjects for each diagnostic findings and final diagnosis are reported)
Main results	16	(*a*) Give unadjusted estimates and, if applicable, confounder-adjusted estimates and their precision (eg. 95% confidence interval). Make clear which confounders were adjusted for and why they were included	YES (all unadjusted prevalence estimates are given; precision was not indicated, but differences between subgroups were tested for statistical significance)
		(*b*) Report category boundaries when continuous variables were categorized	Not applicable (no categorization of continuous variables)
		(*c*) If relevant, consider translating estimates of relative risk into absolute risk for a meaningful time period	Not applicable
Other analyses	17	Report other analyses done—eg analyses of subgroups and interactions, and sensitivity analyses	YES (analysis of clinical chemistry in subgroups is reported)
**Discussion**			
Key results	18	Summarise key results with reference to study objectives	YES (first paragraph of Discussion)
Limitations	19	Discuss limitations of the study, taking into account sources of potential bias or imprecision. Discuss both direction and magnitude of any potential bias	YES (potential selection bias and diagnostic uncertainties are discussed)
Interpreta-tion	20	Give a cautious overall interpretation of results considering objectives, limitations, multiplicity of analyses, results from similar studies, and other relevant evidence	YES (relevant epidemiological studies from Ghana and West Africa are considered)
Generalisa-bility	21	Discuss the generalisability (external validity) of the study results	YES (implications for public health in Ghana are discussed)
**Other information**			
Funding	22	Give the source of funding and the role of the funders for the present study and, if applicable, for the original study on which the present article is based	YES (funders are indicated; there is no original study)

*Give information separately for exposed and unexposed groups.

Note: An Explanation and Elaboration article discusses each checklist item and gives methodological background and published examples of transparent reporting. The STROBE checklist is best used in conjunction with this article (freely available on the Web sites of PLoS Medicine at http://www.plosmedicine.org/, Annals of Internal Medicine at http://www.annals.org/, and Epidemiology at http://www.epidem.com/). Information on the STROBE Initiative is available at www.strobe-statement.org.
